# Long Non-coding RNA Signatures Associated With Liver Aging in Senescence-Accelerated Mouse Prone 8 Model

**DOI:** 10.3389/fcell.2021.698442

**Published:** 2021-07-22

**Authors:** Shuai Zhang, Juanjuan Duan, Yu Du, Jinlu Xie, Haijing Zhang, Changyu Li, Wensheng Zhang

**Affiliations:** ^1^International Cooperation Laboratory of Molecular Medicine, Academy of Chinese Medical Sciences, Zhejiang Chinese Medical University, Hangzhou, China; ^2^Zhuhai Branch of State Key Laboratory of Earth Surface Processes and Resource Ecology, Advanced Institute of Natural Sciences, Beijing Normal University at Zhuhai, Zhuhai, China; ^3^Engineering Research Center of Natural Medicine, Ministry of Education, Faculty of Geographical Science, Beijing Normal University, Beijing, China; ^4^Beijing Key Laboratory of Traditional Chinese Medicine Protection and Utilization, Faculty of Geographical Science, Beijing Normal University, Beijing, China; ^5^Key Laboratory of Vector Biology and Pathogen Control of Zhejiang, School of Medicine, Huzhou University, Huzhou Central Hospital, Huzhou, China; ^6^National and Local United Engineering Research Center for Panax Notoginseng Resources Protection and Utilization Technology, Kunming, China

**Keywords:** long non-coding RNA, liver aging, senescence-accelerated mouse prone 8, senescence-accelerated mouse resistant 1, RNA sequencing

## Abstract

The liver is sensitive to aging because the risk of hepatopathy, including fatty liver, hepatitis, fibrosis, cirrhosis, and hepatocellular carcinoma, increases dramatically with age. Long non-coding RNAs (lncRNAs) are >200 nucleotides long and affect many pathological and physiological processes. A potential link was recently discovered between lncRNAs and liver aging; however, comprehensive and systematic research on this topic is still limited. In this study, the mouse liver genome-wide lncRNA profiles of 8-month-old SAMP8 and SAMR1 models were explored through deep RNA sequencing. A total of 605,801,688 clean reads were generated. Among the 2,182 identified lncRNAs, 28 were differentially expressed between SAMP8 and SAMR1 mice. Gene Ontology (GO) and Kyoto Encyclopedia of Genes and Genomes (KEGG) surveys showed that these substantially dysregulated lncRNAs participated in liver aging from different aspects, such as lipid catabolic (GO: 0016042) and metabolic pathways. Further assessment was conducted on lncRNAs that are most likely to be involved in liver aging and related diseases, such as LNC_000027, LNC_000204E, NSMUST00000144661.1, and ENSMUST00000181906.1 acted on Ces1g. This study provided the first comprehensive dissection of lncRNA landscape in SAMP8 mouse liver. These lncRNAs could be exploited as potential targets for the molecular-based diagnosis and therapy of age-related liver diseases.

## Introduction

Aging is the general decline in physical and mental functioning that occurs in all body cells, tissues, and organs. The world’s elderly population continues to increase at an unprecedented rate, and nearly 17% of the world’s population will reach 65 years or older by 2050 (National Institutes of Health, 2016). Aging has become an increasingly serious problem that imposes a huge financial burden worldwide. Liver is one of the largest organs in the body, and its aging is accompanied by morphological and functional changes. The volume, regenerative ability, and blood flow of the liver reduce remarkably with age (Tajiri and Shimizu, 2013). Elderly liver also shows other characteristics, including cellular mitochondrial injury, hepatocyte nucleus vacuolation, immunologic abnormality, and damaged DNA repair (Lebel et al., 2011; Aravinthan et al., 2012; Poulose and Raju, 2014; Stahl et al., 2018). These changes in liver could lead to serious health issues, such as fatty liver, hepatitis, fibrosis, cirrhosis, and development of hepatocellular carcinoma. Therefore, helping the aging liver to avoid related diseases has become a global concern.

Gene regulation controls how a gene produces necessary protein for a cell to function properly. Dysregulated gene expression in aging liver primarily leads to related diseases. Hu S. J. et al. (2019) reported that liver aging and its related diseases are highly correlated with apoptosis, and the regulatory mechanism of apoptotic gene expression may play an important role in this process. The abnormal expression of genes related to apoptosis, migration, growth, stress response, and immune response may increase the incidence of liver cancer in aging mouse liver (Guo et al., 2009). Strong scientific evidence indicated that the expression of liver mitochondrial genes as regulated by circadian clock becomes altered during aging (Gong et al., 2015). Thus, maintaining normal gene expression and regulation and understanding the underlying molecular mechanisms are necessary to delay liver aging and treat related diseases. The importance of long non-coding RNA (lncRNA) factors in gene regulation has been extensively studied (Chen et al., 2018). With a length of over 200 nucleotides, lncRNA is the best known and most abundant class of non-coding RNAs (ncRNAs) participating in gene transcription at the epigenetic and genetic levels (Gil and Ulitsky, 2019). LncRNAs have multiple functions during aging in different tissues/organs, including liver (He et al., 2018). Jiang and Ning (2020) discussed that lncRNA H19 is widely involved in the pathogenesis of aging liver fibrosis. A series of differentially expressed lncRNAs (Gm12602, Gm12648, Meg3, Rian, and Mirg) contributes to liver aging (White et al., 2015). However, research on lncRNAs associated with liver aging is still in its infancy. Frontier views on lncRNA changes in aging liver, their regulation of gene expression, and their effect on the clinical characteristics and treatment of age-related liver diseases must be developed.

In this study, direct RNA sequencing was used for the expression profiling of lncRNAs in the liver of senescence-accelerated mouse (SAM) prone 8 (SAMP8) and SAM resistant 1 (SAMR1) mice at the 8-month stage. SAMP8 and SAMR1 models were established through the continuous sister-brother breeding of the AKR/J strain based on the phenotypic selection of accelerated aging (Takeda et al., 1981). SAMP8 exhibits characteristics of premature aging (Allison et al., 2020) and shares the characteristics of fatty liver, fibrosis, cirrhosis, and hepatocyte death observed in aged humans (Ye et al., 2004). Thus, this model is reliable for exploring the complexity of liver aging. SAMR1 is resistant to accelerated aging and is widely used as a normal aging control (Wang J. H. et al., 2016). To the best of the author’s knowledge, this research is the first to systematically investigate lncRNAs that are potentially involved in aging liver by using the SAMP8 model. The obtained valuable resources would be helpful for developing therapy against age-related liver disease.

## Materials and Methods

### Animal Model and Tissue Collection

Experimental male SAMP8 (*n* = 25 animals, pathogen- and virus-free) and SAMR1 mice (*n* = 25 animals, pathogen- and virus-free) at the age of 3 months were purchased from Beijing WTLH Biotechnology, Co., Ltd., Beijing, China. The animals were kept in separate cages with standard environment (23°C, 50–60% humidity, and 12 h light/dark cycle) in the laboratory animal center and fed *ad libitum*. The feed weight of the mice was recorded every day, and their body weight was recorded weekly until they reach 8 months. The mice were anesthetized with sodium pentobarbital (50 mg/kg) via intraperitoneal injection (i.p.). Blood was collected through eyeball removal, kept at 4°C temperature for 4 h, and centrifuged at 4000 rpm for 10 min at 4°C for serum collection. The mice were then euthanized through cervical dislocation and dissected to extract their livers. The serum samples were immediately used for blood chemistry test, and the liver tissues were stored in a liquid nitrogen jar at −196°C for RNA sequencing and other experiments.

All animal experiments were conducted in accordance with the “Guide for the care and use of laboratory animals” (National Research Council, 2011) and approved by the Institutional Animal Care and Use Committee of Beijing Normal University (BNU NO. 2020).

### Measurement of Malondialdehyde (MDA) Content

Malondialdehyde (MDA) content was determined following the manufacturer’s instructions (Nanjing Jiancheng Bioengineering Institute, Nanjing, China). The liver samples were weighed and homogenized in phosphate buffered saline, and the supernatant was collected for further analysis. The MDA content was determined via thiobarbituric acid (TBA) reaction following Yun’s method (Yun et al., 2020). The results were expressed in nmol per mg of hepatic tissue protein.

### Blood Chemistry

The mice were deprived of food and water overnight prior to the test. Serum concentrations of total triglycerides (TG), total cholesterol (TC), high-density lipoprotein cholesterol (HDL-C), and low-density lipoprotein cholesterol (LDL-C) were measured using commercially available assay kits (Nanjing Jiancheng Bioengineering Institute, Nanjing, China) in accordance with the manufacturer’s instructions.

### Total RNA Extraction and Quantification

Total RNA was extracted from the liver samples by using TRIzol reagent (Invitrogen, Carlsbad, CA, United States) following the standard protocol proposed by Rio et al. (2010). The extracted RNA was then subjected to a strict quality control. RNA integrity was assessed by 1% agarose gel electrophoresis and the RNA Nano 6000 Assay Kit of the Bioanalyzer 2100 System (Agilent Technologies, Santa Clara, CA, United States), RNA purity was determined using a NanoPhotometer R spectrophotometer (IMPLEN, Westlake Village, CA, United States), and RNA concentration was measured using the Qubit RNA Assay Kit in the Qubit 2.0 Fluorometer (Life Technologies, Carlsbad, CA, United States).

### RNA Sequencing

RNA sequencing was conducted as previously described (Zhang et al., 2016). Six high-quality cDNA libraries were constructed using the total RNAs isolated from liver samples: three for the SAMP8 mice and three for the SAMR1 mice. In brief, 3 μg of high-quality RNA per sample was used as input material. Ribo-Zero rRNA Removal Kit (Epicentre, Madison, WI, United States) was used to remove the ribosomal RNA (rRNA), and ethanol precipitation was conducted to clean up and obtain rRNA-free residues. NEBNext^®^ Ultra^TM^ II Directional RNA Library Prep Kit for Illumina^®^ (NEB, Ipswich, MA, United States) was applied to the rRNA-depleted RNA to generate sequencing libraries. Fragmentation was conducted at 94°C for 15 min in NEBNext First Strand Synthesis Reaction Buffer (5×). Random hexamer primer and M-MuLV Reverse Transcriptase (RNaseH−) were utilized to synthesize the first-strand cDNA. DNA Polymerase I and RNase H were employed to synthesize the second-strand cDNA, which was then purified using 1.8 × Agencourt^®^ AMPure^®^ XP Beads (Beckman Coulter, Brea, CA, United States). NEBNext End Prep Enzyme Mix was used to perform end repair for cDNA library at 20°C for 30 min, followed by 65°C for 30 min. NEBNext Adaptor with hairpin loop structure was ligated to the cDNA using Blunt/TA Ligase Master Mix at 20°C for 20 min. The library fragments measuring 150–200 bp were purified with AMPure XP System. The size-selected and adaptor-ligated cDNA was treated with 3 μl of NEBNext USER^®^ Enzyme at 37°C for 15 min, followed by 5 min at 95°C prior to PCR amplification. PCR was performed with Universal PCR Primer, Index (X) Primer, and Phusion^®^ High-Fidelity DNA Polymerase. The PCR products were purified using the AMPure XP system, and library quality was assessed by the Bioanalyzer 2100 system (Agilent Technologies, Santa Clara, CA, United States). TruSeq PE Cluster Kit v3-cBot-HS (Illumina, San Diego, CA, United States) was employed to cluster the index-coded samples. The libraries were forwarded to a sequencing run on the Illumina HiSeq 4000 system at the Novogene Bioinformatics Institute (Beijing, China) to generate 150 bp paired-end reads.

### Transcriptome Assembly

High-quality clean reads were obtained using in-house perl scripts by removing the reads with adaptors, low-quality reads, and reads poly-N >10% from raw data. The GC%, Q30, and Q20 of the clean data were calculated. GRCm38 was used as a mouse reference genome, and the paired-end clean reads were mapped to this reference sequence^1^ using TopHat v.2.0.9 (Kim et al., 2013). The mapped reads of each sample were assembled to transcripts by running Cufflinks v2.1.1 software (Trapnell et al., 2010).

### LncRNA Identification and Conservation Analysis

The pipeline for lncRNA discovery and identification was prepared as previously described (Zhang et al., 2016). Transcripts with <200 bp in length, less than two exons, and less than three reads coverage were first removed. The remaining transcripts were then compared with known mouse lncRNAs, mRNAs, snoRNA, snRNA, pre-miRNA, tRNA, rRNA, and pseudogenes and then assessed using CNCI, CPC, PFAM, and phyloCSF software (Kong et al., 2007; Mistry et al., 2007; Lin et al., 2011; Sun et al., 2013). Finally, the qualifying lncRNAs were selected and designated as novel or known lncRNAs.

PhyloFit and phastCons from Phast package were used to compute a set of conservation scores for lncRNAs and protein-coding transcripts (Siepel et al., 2005).

### Expression Analysis

Long non-coding RNA and mRNA expression levels in each sample were measured as fragments per kilobase of exon model per million mapped fragments (FPKM) by using the Cuffdiff. Transcripts with *p* adjust value < 0.05 (*q* value < 0.05) were considered as statistically significant between SAMP8 and SAMR1.

### Target Prediction

Long non-coding RNAs may affect the gene expression by playing *cis* and *trans* regulating roles (Guil and Esteller, 2012; Yan et al., 2017). Here, only the differentially expressed lncRNAs and mRNAs were used in the prediction to explore the potential function of lncRNAs. The mRNAs within 100 kb upstream and downstream of lncRNAs were selected as *cis* results. For the *trans* role, the lncRNAs identify each other based on expression levels, and the absolute values of Pearson’s correlation coefficients >0.95 (|r| > 0.95) were selected.

### Gene Ontology (GO) and Kyoto Encyclopedia of Genes and Genomes (KEGG) Surveys

Gene ontology (GO) and Kyoto Encyclopedia of Genes and Genomes (KEGG) enrichment analyses were applied to the differentially expressed protein-coding genes and target genes of lncRNAs. GOseq R package was used to perform GO analysis. GO terms with *p* value < 0.05 were recognized as significantly enriched. KOBAS software was used to detect the enriched KEGG pathways. Hypergeometric *p* value < 0.05 was considered significant.

### Real-Time qPCR Validation

qPCR was performed on a 7500 Real-time PCR machine (Applied Biosystems, Foster City, CA, United States) by using the SYBR Green qPCR Kit (GenePharma, Shanghai, China) following the manufacturer’s protocol. The 20 μl reaction volume contained 8.2 μl of H_2_O, 0.4 μl of each primer, 1 μl of cDNA, and 10 μl of 2 × SYBR Green qPCR Mix. The conditions were as follows: 95°C for 5 min, followed by 40 cycles (95°C for 15 s and 60°C for 30 s). Specific quantitative primers were designed using the primer-BLAST tool on the NCBI website as listed in Table 1. Mouse housekeeping gene β-actin was selected as an internal control. Each experiment was performed in triplicate.

**TABLE 1:** Primers used in qPCR analysis.

**Acession No.**	**Primer sequence (5′-3′)**
ENSMUST00000144661.2	F: GCTGGCCTGACAGGTAGGTA R: TGGCAACCTTCCAAAGCTGA
ENSMUST00000180730.3	F: AAGGACGCATTTCTCCCTCG R: TCCAGTCAAGTCGTGAAGGC
ENSMUST00000181906.1	F: TTGTTTGCCCTACCTGTCCC R: GATCGATCACCAGGCAACCT
LNC_000366	F: TCCTGGGAGTTGGAGTTCTG R: CACCAGCACCTGGCTTATTT
LNC_000204	F: AGGCATCAAGCAAATGGGGA R: AACACTCTTGCTGCGCTAGT
LNC_000150	F: ACTCAACTTCCTTCATTCCAGGT R: CCCATCTCATGAGGGTGCTC
LNC_000027	F: GCAGAGAGGCTGTTCTACGG R: GGCTGGTCGTAGCTCATGTT
LNC_000426	F: GATGGTACTGAGAACGGGCA R: GGCTTGTAAGGCATGGAAGTG
ENSMUST00000095147	F: GATACCGGCCTAGGAAAGCC R: AAGGCCATGGTCAACTGAGG
ENSMUST00000119103	F: CCAGTGGCTGCAGATTCAGA R: TGTCAAGAAGGCTGCTGAGG
ENSMUST00000007253	F: AGGAGCCGATGTACTTACTGG R: CTGCTTGCTCCTTTCTCACAG
ENSMUST00000004136	F: CTCCAGCTCAACCTACCTGAA R: CACTGGCTTCGGTCAAAGTT
ENSMUST00000108357	F: ATGAGAAGCGGCTGTCTAGC R: CCAGCACCGTCACCTCATAA
ENSMUST00000167487	F: ACGGCCAGTGTGTTCCTAAC R: GCGCCACAGCTGATTTCATT
ENSMUST00000102632	F: CCCTTGGAGAGTGACAGCAG R: GGGACTAGAGGTAAGGCCCA
ENSMUST00000044602	F: TAGTGGGCAGGATTTCAGGC R: AGCTCTGGGGTCTAGTGAGG
ENSMUST00000026211	F: CGCATGGAGCTCTTCCTGTT R: TCAAGGTCCTTTGGCTCCAC
ENSMUST00000118365	F: AGGAGCCCAACAAACAACAC R: GAGGCTGGAAGTGTTTGCTC
β-Actin	F: TACAATGAGCTGCGTGTGGC R: CAGCACTGTGTTGGCATAGAGGT

*LNC_000XXX means novel lncRNA.*

### Statistical Analysis

Data were analyzed using Graph pad prism 8 software and represented as mean ± standard error of the mean (SEM). Body weight data were compared by applying two-way repeated measures ANOVA. Two-tailed unpaired Student’s *t*-test was employed to compare other types of data. Statistical significance was accepted at *p* value < 0.05.

## Results

### Body Weight, Liver Weight, Food Intake, Liver MDA Contents, and Serum Lipid Levels in the SAMP8 and SAMR1 Mouse Models

**FIGURE 1:**
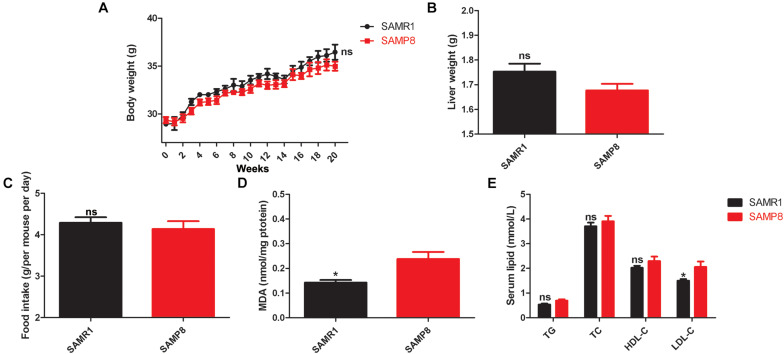
Variability in body weight, liver weight, food intake, liver MDA contents, and serum lipid levels in SAMP8 mice. Mean values of **(A)** weekly body weight, **(B)** liver weight, **(C)** daily food intake, **(D)** MDA contents, and **(E)** serum lipid levels between the SAMP8 and SAMR1 mice. The data were presented as the mean ± SEM. Body weight data were analyzed by two-way repeated measures ANOVA. Other types of data were analyzed by two-tailed unpaired Student’s *t*-test. *n* = 10. *p value < 0.05 vs. SAMP8, ns means non-significant.

Figures 1A–C show no remarkable difference in the body weight, liver weight, and food intake between SAMP8 and SAMR1 mice at 8 months of age (*p* value > 0.05). This finding implied that aging does not alter the body weight, liver weight, and food intake of these mice. Oxidative stress marker MDA is one of the major end products of lipid peroxidation and is traditionally used as an indicator of liver tissue damage (Wang et al., 2019). Figure 1D shows that the levels of liver MDA in 8-month-old SAMP8 mice were significantly increased compared with those in SAMR1 mice (*p* value < 0.05). In addition, the serum LDL-C levels of SAMP8 mice were dramatically higher than those of SAMR1 mice (*p* value < 0.05, Figure 1E). No differences in TG, TC, and HDL-C were found between the two groups (*p* value > 0.05, Figure 1E). These findings revealed that aging is a potential cause of impaired hepatic cholesterol synthesis and metabolism, abnormal function of low-density lipoprotein receptor, and even fatty liver diseases. The livers of the 8-month-old SAMP8 mice initially exhibited impairment on oxidative stress and lipid metabolism, the common clinical characteristics of aging human liver.

### RNA Sequencing Roundup

A total of 628,530,304 raw reads were generated using the Illumina HiSeq 4000 system. After the low-quality reads were removed, 605,801,688 clean reads were obtained. The high-quality clean reads were mapped to the mouse reference genome sequence by TopHat v.2.0.9. The mapping rates were approximately 87.11 and 84.01% in SAMP8 and SAMR1 mice, respectively. In accordance with the Cufflinks v2.1.1 results, 137,750 transcripts were assembled and used for the following analysis.

### Identification of LncRNAs and mRNAs in SAMP8 and SAMR1 Mouse Livers

**FIGURE 2:**
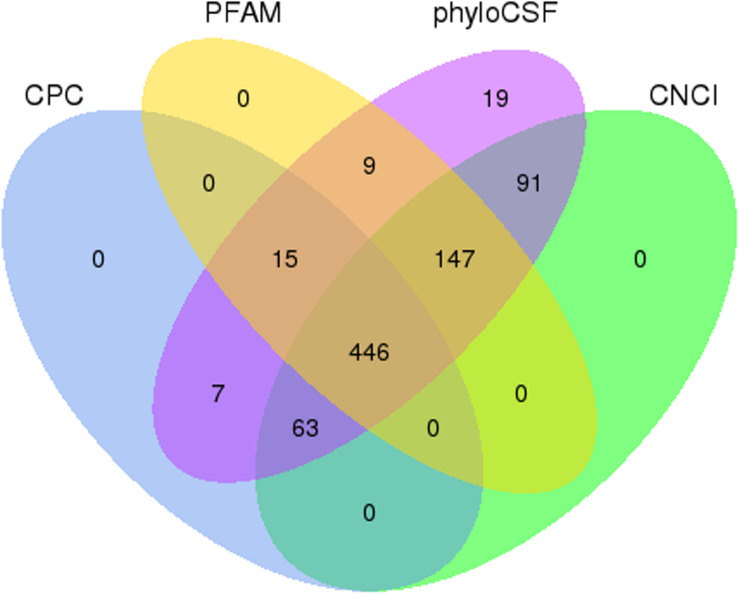
Coding potential screening. Venn diagram containing CPC, CNCI, PFAM, and phyloCSF data used to analyze the coding potential of presumed lncRNAs. The data shared by the four tools were designated as novel lncRNAs for subsequent analyses.

After the initial screening, 1,736 known mouse lncRNAs corresponding to 1,399 lncRNA genes and 797 presumed lncRNAs were detected. CNCI, CPC, PFAM, and phyloCSF tools were used to further confirm these 797 presumed lncRNAs. A total of 446 novel lncRNAs (defined as LNC_000XXX) corresponding to 317 lncRNA genes were identified (Figure 2). In addition, 54,275 mRNA transcripts corresponding to 21,959 protein-coding genes were also detected. In summary, 2,182 lncRNAs and 54,275 mRNAs were detected in SAMP8 and SAMR1 mouse livers.

### Comparison of LncRNA and mRNA Features

**FIGURE 3:**
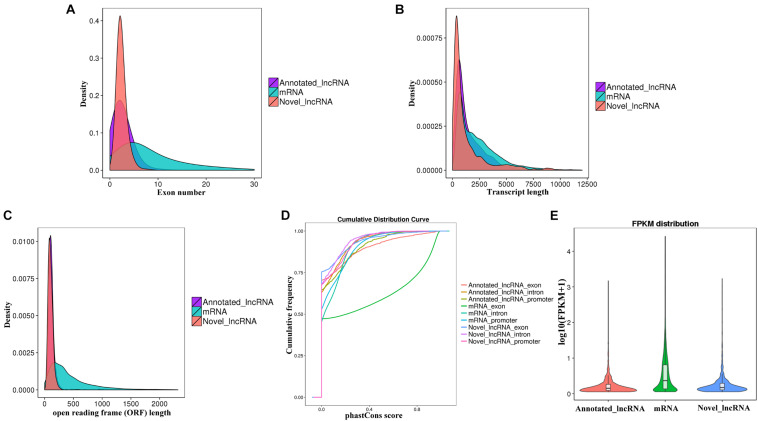
Feature comparison for the identified lncRNAs and mRNAs. Distribution of **(A)** number of exons, **(B)** transcript lengths, **(C)** open reading frame (ORF) lengths in the mRNAs and lncRNAs. **(D)** conservation analysis of sequence, and **(E)** expression level analysis in the mRNAs and lncRNAs.

The characteristics of the obtained 2,182 lncRNAs and 54,275 mRNAs were analyzed. Most of the known and novel lncRNAs contained fewer exons than mRNAs (Figure 3A). The average full length and open reading frame (ORF) length of the known and novel lncRNAs were shorter than those of mRNAs (Figures 3B,C). The conservatism of the known and novel lncRNAs was significantly lower than that of mRNAs (Figure 3D). In addition, the known and novel lncRNAs had significantly lower expression than mRNAs (Figure 3E). These results are consistent with previous studies (Singh et al., 2017; Wu et al., 2018).

### Differential Expression of LncRNAs and mRNAs Between SAMP8 and SAMR1 Mouse Livers

**FIGURE 4:**
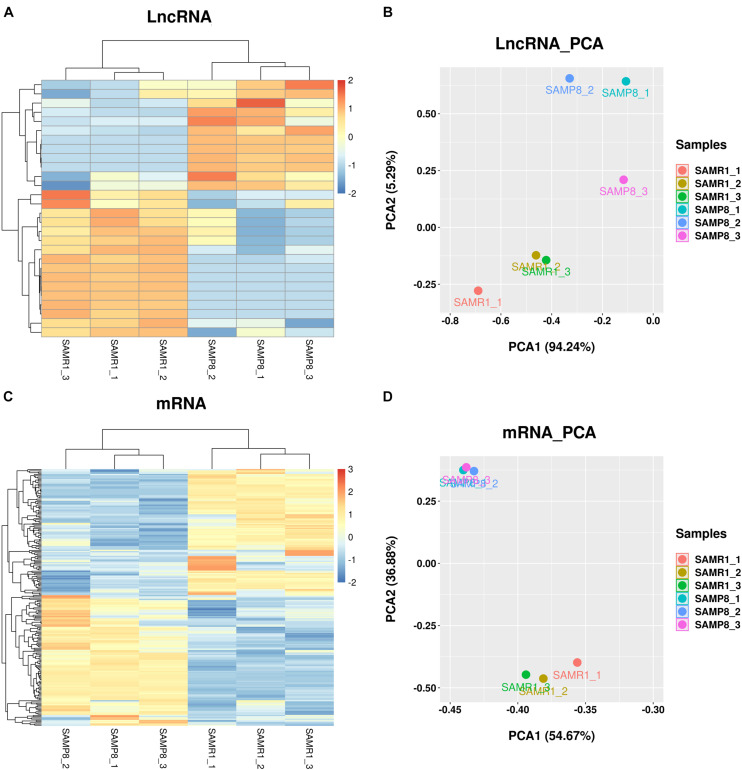
Cluster analysis and principal component analysis (PCA) of differentially expressed lncRNAs and mRNAs in the SAMP8 and SAMR1 mice. **(A,C)** Cluster analysis. **(B,D)** Principal component analysis.

FPKM was used to estimate the expression of lncRNA and mRNA transcripts. Among the 28 significantly dysregulated lncRNA transcripts (corresponded to 28 lncRNA genes) identified between the two groups (Table 2, *q* value < 0.05), 12 were upregulated and 16 were downregulated in SAMP8 mice. Among the 210 remarkably dysregulated mRNA transcripts (corresponding to 205 protein-coding genes) identified, 107 were upregulated and 103 were downregulated in SAMP8 mice (Supplementary Table 1, *q* value < 0.05). Cluster analysis and principal component analysis (PCA) were performed for the 28 differentially expressed lncRNAs (Figures 4A,B) and 210 significantly dysregulated mRNAs (Figures 4C,D). The three replicates of the SAMP8 group were clustered together, and the same was performed on the SAMR1 group.

**TABLE 2:** Significantly differentially expressed lncRNAs in SAMP8 and SAMR1 mice.

**Transcript_id**	**Gene_Symbol**	**SAMP8 FPKM**	**SAMR1 FPKM**	**log2 (fold change)**	***q* value**
LNC_000052	No official symbol	0.82	0	inf	0.004
LNC_000150	No official symbol	0.34	1.04	−1.6312	0.004
LNC_000284	No official symbol	0.37	0	inf	0.004
LNC_000350	No official symbol	0	0.36	#NAME	0.004
LNC_000358	No official symbol	1.42	0.27	2.3861	0.008
ENSMUST00000144661.2	Rin2	0.10	0.82	−3.08267	0.004
ENSMUST00000181142.2	9030616G12Rik	9.55	3.65	1.38847	0.038
LNC_000027	No official symbol	0	0.43	#NAME	0.004
ENSMUST00000156081.2	Gm12840	36.42	115.50	−1.66502	0.004
ENSMUST00000205674.2	Gm38832	0	2.91	#NAME	0.004
LNC_000168	No official symbol	0.24	2.71	−3.52268	0.004
ENSMUST00000181620.2	D930016D06Rik	1.27	3.09	−1.28122	0.026
ENSMUST00000180730.3	9930014A18Rik	1.66	6.29	−1.91974	0.004
LNC_000204	No official symbol	0	1.16	#NAME	0.004
LNC_000207	No official symbol	0.41	0	inf	0.004
ENSMUST00000181242.1	Gm26870	9.94	2.45	2.0188	0.004
LNC_000374	No official symbol	2.18	0.19	3.54443	0.021
ENSMUST00000128160.2	4930481B07Rik	3.15	0.80	1.97526	0.021
ENSMUST00000181906.1	Cep83os	0.49	1.83	−1.91792	0.004
LNC_000341	No official symbol	0.87	0	inf	0.015
ENSMUST00000188595.2	1810064F22Rik	9.42	4.51	1.06251	0.008
ENSMUST00000153865.2	Gm13775	32.17	8.05	1.99912	0.004
LNC_000366	No official symbol	0	1.60	#NAME	0.004
ENSMUST00000190861.2	Gm28068	0.08	0.37	−2.26699	0.042
LNC_000426	No official symbol	0	1.24	#NAME	0.004
LNC_000435	No official symbol	0.20	0.69	−1.82391	0.031
ENSMUST00000180863.1	Gm26809	19.03	63.71	−1.74322	0.004
ENSMUST00000181253.10	Rian	6.69	3.01	1.15408	0.049

*Log2 transformation is applied to simplify the fold change values. inf means no expression in SAMR1 mice.*

*#NAME means no expression in SAMP8 mice. LNC_000XXX means novel lncRNA.*

### Functional Enrichment Analysis Revealing the Close Correlation Between LncRNAs and Liver Aging

*Cis* and *trans* analyses were performed to predict the underlying relationships between altered lncRNAs and mRNAs. With 100 kb as the cutoff in *cis*, 2 out of 28 differentially expressed lncRNA genes corresponded to two protein-coding genes (Supplementary Table 2). According to Pearson’s correlation coefficients (|r| > 0.95) in *trans*, 12 out of 28 differentially expressed lncRNA genes corresponded to 41 protein-coding genes (Supplementary Table 2). The abovementioned target genes were subjected to GO and KEGG analyses. A total of 147 GO terms (Supplementary Table 3, *p* value < 0.05) and 26 KEGG pathways (Supplementary Table 4, *p* value < 0.05) were significantly enriched. The top five GO terms were arachidonic acid epoxygenase activity (GO: 0008392), oxidoreductase activity (GO: 0016491), chitin catabolism (GO: 0006032), intracellular membrane-bounded organelle (GO: 0043231), and icosanoid biosynthesis (GO: 0046456). The top five KEGG pathways were retinol metabolism, inflammatory mediator regulation of TRP channels, metabolic pathways, phagosome, and allograft rejection. Several liver aging-associated terms and pathways were also observed, such as oxidoreductase activity (GO: 0016491), lipid catabolism (GO: 0016042), steroid hydroxylase activity (GO: 0008395), retinol metabolism, and metabolic pathways. Therefore, lncRNAs could regulate protein-coding genes associated with liver aging through their *cis* and *trans* roles.

Gene Ontology and KEGG surveys were also performed on 205 significantly dysregulated protein-coding genes. A total of 237 GO terms (Supplementary Table 5, *p* value < 0.05) and 33 KEGG pathways (Supplementary Table 6, *p* value < 0.05) were significantly enriched. The top five GO terms were oxidoreductase activity (GO: 0016491), arachidonic acid epoxygenase activity (GO: 0008392), heme binding (GO: 0020037), oxidoreductase activity, acting on paired donors, with incorporation or reduction of molecular oxygen (GO: 0016705), and oxidation–reduction (GO: 0055114). The top five KEGG pathways were retinol metabolism, metabolic pathways, steroid hormone biosynthesis, arachidonic acid metabolism, and chemical carcinogenesis. These results were reflected in liver aging, such as several GO terms (e.g., GO: 0016491, GO: 0055114, and GO: 0004806) and KEGG pathways (e.g., retinol metabolism, metabolic pathways, and fatty acid degradation).

### Functional Specificities of LncRNAs in Liver Aging

**FIGURE 5:**
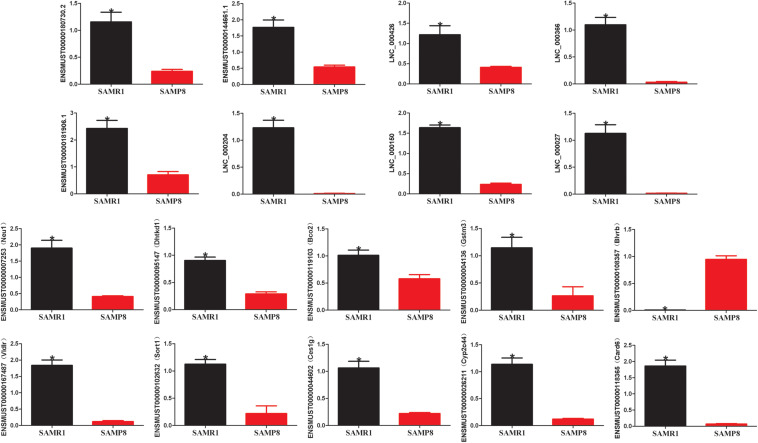
Validation of lncRNA and mRNA expression by quantitative polymerase chain reaction (qPCR). β-actin gene was used as a housekeeping internal control. The relative expression of each transcript was represented as mean ± SEM. Data were analyzed by two-tailed unpaired Student’s *t*-test. *n* = 3 [three mice per group (biological replicates), three times per mouse (technical replicates)]. *p value < 0.05 vs. SAMP8.

Two limiting conditions were imposed to deeply understand the relationship of lncRNAs with liver aging and related diseases. The first was that the lncRNAs and their target genes must be differentially expressed in the liver tissues of SAMP8 and SAMR1 mice, and the other was the selected pairs (lncRNA–mRNA) must be associated with liver aging and related diseases. A pair matching the above two conditions would be selected. Ces1g reduces hepatic steatosis and counteracts dyslipidemia (Bahitham et al., 2016) and is regulated by LNC_000027, ENSMUST00000144661.2, ENSMUST00000181906.1, and LNC_000204. Bco2 maintains normal hepatic lipid and cholesterol homeostasis (Lim et al., 2018) and is acted upon by LNC_000027 and LNC_000204. Neu1, a gene involved in hepatic glucose and lipid metabolism (Hu Y. et al., 2019), is targeted by ENSMUST00000144661.2, LNC_000366, and LNC_000150. Dhtkd1 possibly modulates mitochondrial biogenesis, enhances energy expenditure to control liver steatosis (Lim et al., 2014), and is acted upon by LNC_000204, LNC_000150, ENSMUST00000144661.2, and ENSMUST00000181906.1. Card6 protects against hepatic steatosis by suppressing Ask1 (Sun et al., 2018) and is regulated by ENSMUST00000180730.3. Gstm3 is an anti-oxidative gene that acts against liver injury (Sun et al., 2017; Zhao et al., 2017) and is controlled by ENSMUST00000181906.1, LNC_000204, and LNC_000150. VLDLR plays an important role in hepatic steatosis (Zarei et al., 2018) and is governed by LNC_000027 and LNC_000426. The proliferation of hepatocellular carcinoma cells is inhibited by the knockdown of BLVRB (Huan et al., 2016), which is regulated by LNC_000204. Sort1 overexpression in mouse liver reduces plasma triglycerides and cholesterol (Strong et al., 2012; Conlon, 2019). Cyp2c44, a member of cytochrome P450 (P450), mediates arachidonic acid metabolism and further regulates inflammation in hepatic tissues (Theken et al., 2011). Sort1 and Cyp2c44 are targeted by LNC_000150. The detailed results are listed in Table 3. The expression of these selected pairs was examined by qPCR to further prove their reliability. As shown in Figure 5, all the mRNA and lncRNA transcripts were detected and exhibited significantly different expression between the SAMP8 and SAMR1 mice. The qPCR results were consistent with the RNA sequencing data. Therefore, these lncRNAs are most likely to participate in liver aging and related diseases.

**TABLE 3:** Most possibly involved lncRNAs in liver aging and related diseases.

**LncRNAs**	**log2 fold change (SAMP8 vs. SAMR1)**	**Potential target protein-coding genes**
ENSMUST00000144661.2	−3.08267	Ces1g, Neu1, Dhtkd1
ENSMUST00000180730.3	−1.91974	Card6
ENSMUST00000181906.1	−1.91792	Ces1g, Dhtkd1, Gstm3
LNC_000027	#NAME	Ces1g, Bco2, VLDLR
LNC_000204	#NAME	Ces1g, Bco2, BLVRB, Gstm3, Dhtkd1
LNC_000366	#NAME	Neu1
LNC_000150	−1.6312	Cyp2c44, Sort1, Neu1, Gstm3, Dhtkd1
LNC_000426	#NAME	VLDLR

*Log2 transformation is applied to simplify the fold change values. #NAME means no expression in SAMP8 mice. LNC_000XXX means novel lncRNA.*

## Discussion

With the rapid development of high-throughput sequencing technologies, remarkable headway has been achieved in the field of ncRNAs. In mammals, over 90% of the genomes are transcribed as ncRNAs (Jathar et al., 2017). LncRNAs are a large class of ncRNAs that have become a research hotspot. Owing to their regulating role for protein-coding genes at the epigenetic, transcriptional, and post-transcriptional levels (Statello et al., 2021), lncRNAs are hypothesized to participate in many physiological and pathological processes. One of which is aging, the greatest contributor to liver function failure and is associated with liver diseases, such as fatty liver, hepatitis, fibrosis, cirrhosis, and hepatocellular carcinoma (Kim et al., 2015). Recent works have shifted focus to the regulatory effects of lncRNAs on liver aging and related diseases (White et al., 2015; Jiang and Ning, 2020); however, these efforts remain lacking. The present study aimed to investigate the lncRNAs involved in liver aging and related diseases through deep RNA sequencing. the difficult access to the tissue samples of aging human liver, an appropriate animal model was employed as an alternative. SAMP8, a widely used aging model, displays pathological changes in the liver that closely mimic those in aging human liver (Ye et al., 2004). Blood chemistry and detection kit test confirmed that the 8-month-old SAMP8 mice exhibited liver impairments, including oxidative stress and lipid metabolism disorder. The findings in this study could provide insights into the changes in the expression profile of lncRNAs during liver aging and serve as reference for diagnosis and treatment strategies for age-related liver diseases.

A single gene can be processed to create multiple transcript isoforms, and this process is referred to as alternative splicing. This pattern also widely exists in lncRNA genes that regulate protein-coding in many physiological and pathological processes (Chen et al., 2014; Shahryari et al., 2015). In this study, 2,182 lncRNA transcripts corresponded to 1,716 lncRNA genes that exhibited alternative splicing events. This finding may have functional importance in liver aging and related diseases. The 2,182 identified lncRNAs had shorter full length and ORF length, less conservation, fewer exons, and lower expression levels than mRNAs. These features were shared in other mammals, including humans, goats, and pigs (Derrien et al., 2012; Ren et al., 2016; Wang Y. Y. et al., 2016). This commonality in mammals possibly reflects the importance of lncRNA in gene regulation, control, and guidance. The RNA sequencing data generated 54,275 mRNAs (corresponding to 21,959 protein-coding genes) for the two groups. These mRNA transcripts were used to build the functional networks of lncRNA–mRNA involved in the regulation of liver aging and related diseases.

Expression alteration is usually utilized to understand the physiological or pathological differences between two species. Statistics analysis revealed 28 dysregulated lncRNAs between the two groups. Different from mRNAs, a large proportion of lncRNAs had extremely low abundance, and the copy number of many lncRNAs was even less than one per cell (Mercer et al., 2011). Most of these differentially expressed lncRNAs showed a FPKM of less than 5 (Table 2), such as LNC_000052, LNC_000150, ENSMUST00000144661.2, ENSMUST00000205674.2, ENSMUST00000181620.2, ENSMUST00000181906.1, LNC_000350, LNC_000358, LNC_000284, and ENSMUST00000190861.2. These scarce lncRNAs might play important roles in triggering downstream effects (Mercer et al., 2009; Clark et al., 2011; Seiler et al., 2017). Their low abundance is often associated with relatively small changes in their expression (Table 2), such as LNC_000284 (SAMP8 FPKM: 0.37; SAMR1 FPKM: 0) and ENSMUST00000190861.2 (SAMP8 FPKM: 0.08; SAMR1 FPKM: 0.37). Regardless, these lncRNAs may still have effects on liver aging. Not all the 28 dysregulated lncRNAs might have functions in liver aging; some could produce a response or compensation. A total of 210 significantly dysregulated mRNA transcripts were also identified.

Long non-coding RNA regulation falls into two common types: *cis*- and *trans*-acting (Yan et al., 2017). The 28 lncRNAs and 210 mRNAs were applied to predict the regulatory networks of lncRNA–mRNA. The results identified 42 target protein-coding genes. One lncRNA had the ability to regulate multiple protein-coding genes, e.g., ENSMUST00000144661.2 adjusts the expression of Ces1g, Neu1, and Dhtkd1 genes. By contrast, one protein-coding gene could be controlled by several lncRNAs, e.g., Gstm3 is targeted by ENSMUST00000181906.1, LNC_000204, and LNC_000150. These findings indicated the complexity of lncRNA functional regulation in liver aging and related diseases. Specific GO and KEGG analyses were performed on the 42 lncRNA targeting genes. Some Go terms and KEGG pathways were pertaining to liver aging and age-related liver diseases, such as lipid catabolism (GO: 0016042), retinol metabolism, and metabolic pathways. The retinol metabolism pathway was selected for specific illustration. Retinol is a form of retinoid, a derivative of vitamin A. Approximately 70–90% of retinoids are stored in hepatic stellate cells (Shirakami et al., 2012). Alterations in retinoid metabolism and release by hepatic stellate cells contribute to liver damage, including fatty liver and hepatic fibrosis (Romeo and Valenti, 2016; Shiota, 2017). A close link was observed between the dysregulated lncRNAs and liver aging and related diseases. The lncRNA–mRNA pairs that were possibly involved in the regulation of liver aging and related diseases were also filtered (Table 3). qPCR was used to validate the accuracy of the above-mentioned lncRNA–mRNA pairs. All qPCR results were consistent with the RNA sequencing data. Therefore, the above lncRNA–mRNA networks in liver aging and related diseases are highly complex, diverse, and reliable.

White et al. (2015) performed RNA-sequencing to explore differences in lncRNA expression in the liver of young (4-month-old) and aged (28-month-old) BALB/c mice and detected 43 upregulated and 16 downregulated lncRNAs. Among which, only lncRNA Rian (ENSMUST00000181253.10) shared an overlap, and its expression was higher in the aged mice (8-month-old SAMP8 and 28-month-old BALB/c) than in controls (8-month-old SAMR1 and 4-month-old BALB/c). Their results are quite different from the current RNA-sequencing data possibly due to disparities in strain and age. SAMP8 is a naturally occurring mouse strain that displays a phenotype of accelerated aging and an age-associated decline in liver function (Takeda et al., 1981; Ye et al., 2004). Although the SAMP8 mice exhibit liver damages that commonly occur with aging, they cannot fully reflect normal liver aging process. Moreover, the detailed molecular mechanisms underlying the functions of these lncRNAs in liver aging and age-related liver diseases are not fully elucidated. Our ongoing studies will strive to clarify these results, and this endeavor will be an enormous challenge for many years to come.

In conclusion, this study elucidated the liver lncRNA profiles of 8-month-old SAMP8 and SAMR1 mice. Several functional lncRNA–mRNA pairs that are involved in liver aging were detected and exploited to be potential therapeutic targets or diagnostic biomarkers for age-related liver diseases.

## Data Availability Statement

The RNA sequencing raw data reported in this manuscript has been deposited in the NCBI Sequence Read Archive (SRA). The accession number is SRP323256. The link is https://www.ncbi.nlm.nih.gov/Traces/study/?acc=PRJNA735929.

## Ethics Statement

The animal study was reviewed and approved by The Institutional Animal Care and Use Committee of Beijing Normal University.

## Author Contributions

SZ, CL, and WZ designed the experiments and contributed to the reagents, materials, and analysis tools. SZ and JD performed the experiments. SZ, JD, YD, JX, and HZ analyzed the data. SZ wrote the manuscript. All authors read and approved the final manuscript.

## Conflict of Interest

The authors declare that the research was conducted in the absence of any commercial or financial relationships that could be construed as a potential conflict of interest.

## Footnotes

1 ftp://ftp.ensembl.org/pub/release-95/gtf/mus_musculus/

## Supplementary Material

Supplementary Table 1 Significantly differentially expressed mRNA transcripts in SAMP8 and SAMR1 mice.

Supplementary Table 2 Target prediction of significantly differentially expressed lncRNAs in *cis* (100K) and *trans*.

Supplementary Table 3 GO enrichment analysis of the *cis* and *trans* target genes.

Supplementary Table 4 KEGG enrichment analysis of the *cis* and *trans* target genes.

Supplementary Table 5 GO enrichment analysis of the significantly dysregulated protein-coding genes.

Supplementary Table 6 KEGG enrichment analysis of the significantly dysregulated protein-coding genes.
